# Inner Retinal Layer Thickness Alterations in Early Age Related Macular Degeneration in Eyes with Subretinal Drusenoid Deposits or Conventional Drusen

**DOI:** 10.3390/jcm10215136

**Published:** 2021-10-31

**Authors:** Solmaz Abdolrahimzadeh, Mariachiara Di Pippo, Edoardo Sordi, Sandrine Anne Zweifel

**Affiliations:** 1 Ophthalmology Unit, Neurosciences, Mental Health, and Sense Organs (NESMOS) Department, Faculty of Medicine and Psychology, University of Rome Sapienza, St. Andrea Hospital, Via di Grottarossa 1035/1039, 00189 Rome, Italy; mariachiara.dipippo@gmail.com (M.D.P.); edosordi@hotmail.com (E.S.); 2 Department of Ophthalmology, University Hospital Zurich, Frauenklinikstrasse 24, 8091 Zurich, Switzerland; sandrine.zweifel@usz.ch; 3 University of Zurich, Rämistrasse 71, 8006 Zurich, Switzerland

**Keywords:** subretinal drusenoid deposits, reticular pseudodrusen, conventional drusen, age related macular degeneration, inner retina

## Abstract

The purpose of this study was to evaluate central and parafoveal inner retinal layer thickness in patients with subretinal drusenoid deposits (SDD) or conventional drusen (CD). Participants underwent comprehensive ophthalmoscopic examination. Evidence of SDD or CD was evaluated with near infrared reflectance and spectral domain optical coherence tomography. Quantification of subfoveal lesions was made through a qualitative analysis of vertical and horizontal SD-OCT scans centered on the fovea. Inner retinal layer macular thickness measurements were obtained for central circles with 1, 3, and 5 mm diameter. Continuous variables were compared by the analysis of covariance (ANCOVA) with post-hoc Tukey HSD correction for multiple comparison analysis. Fifty-five patients were included in the study; 18 eyes with SDD alone, 19 eyes with CD alone, and 18 eyes of healthy age-matched subjects. Eight eyes with SDD (44%) and 13 eyes with CD (68%) had subfoveal lesions. There was significant reduction in the inner retinal layer thickness in the central 1mm area and in the superior 3 mm area in the SDD and CD group compared to controls. In conclusion the inner retinal layer is thinner in the central macula and in the superior parafovea in eyes.

## 1. Introduction

Late manifestations of age related macular degeneration (AMD) are the primary pathology responsible for irreversible loss of vision in developed countries. The principal hallmarks of AMD are drusen that are focal deposits of extracellular material beneath the retinal pigment epithelium [1]. Growing evidence shows that subretinal drusenoid deposits (SDD), otherwise called reticular pseudodrusen, are an important phenotype in AMD. These deposits are located in the subretinal space, between the photoreceptors and retinal pigment epithelium layer with different retinal topography with respect to drusen as they are localized predominantly in the superior macula outside of the fovea, and to a less extent in the foveal region, whereas soft drusen are characteristically found in the central macula [2,3]. Classically AMD is characterized by outer retinal involvement [4,5] however, recent evidence has also shown inner retinal layer changes [6,7,8]. Thinning of the inner retinal structures has been reported for the retinal nerve fiber layer (RNFL), ganglion cell layer (GCL), inner plexiform layer (IPL), and ganglion cell complex (GCC) [9,10,11,12]. Suggested pathogenetic theories for inner retinal involvement are microvascular alterations with inner retinal ischemia and post-receptor trans-synaptic degeneration [1,6,7,11,13,14]. SDD are associated with both forms of late AMD, geographic atrophy and neovascularization [15]. In eyes with SDD there is substantial literature on the outer retinal layers and the choroid [16,17,18]. Only two reports describe the inner retinal changes in eyes with SDD where thinning of the GCL and IPL was shown [19,20]. Although conventional drusen (CD) and SDD are frequently found simultaneously in eyes with AMD [21,22] with a prevalence of one or the other, the ideal model of study would be to stratify and separately analyze eyes with either one or the other type of deposits alone. Therefore, in the present report we used strict inclusion and exclusion criteria to select patients and assess inner layer thickness in the central and parafoveal macular area among eyes with pure SDD, pure CD, and eyes of healthy age-matched subjects.

## 2. Materials and Methods

**Figure 1:**
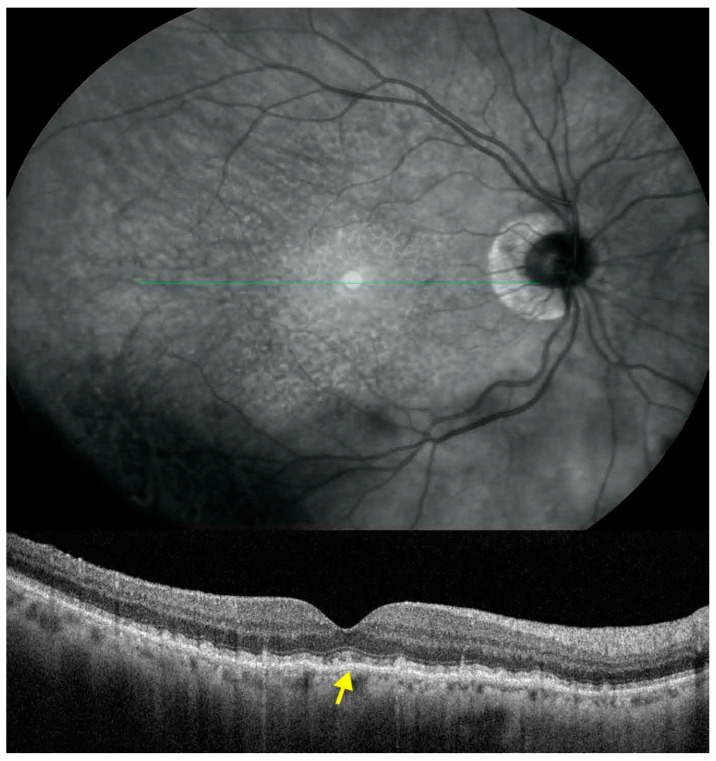
Spectral domain optical coherence tomography (SDOCT) scan of an eye with subfoveal subretinal drusenoid deposits (SDD). The SDOCT cross-sectional scan shows hyperreflective material between the retinal pigment epithelium and the ellipsoid zone with focal changes of the latter. SDD located under the fovea is shown with the arrow.

This was an observational cross-sectional study including 55 eyes of 55 subjects (18 eyes with SDD, 19 eyes with CD, and 18 eyes of healthy age-matched subjects) carried out at the Retina Centre of the Ophthalmology Unit of the University of Rome Sapienza, St. Andrea Hospital. The study had Institutional Review Board Approval from the University of Rome, Sapienza. All the procedures were performed in agreement with the principles of the declaration of Helsinki. Informed consent was obtained from the subjects after explanation of the nature of the study. Inclusion criteria were diagnosis of early/intermediate AMD in patients with age above 50 years and near infrared reflectance (NIR) and spectral domain optical coherence tomography (SDOCT) evidence of CD (defined as the CD group) or SDD (defined as the SDD group). Healthy age-matched subjects were included as the control group and were patients who underwent routine ophthalmological examination in our general ophthalmology clinic. Exclusion criteria were spherical equivalent above 4 Diopters; glaucoma or intraocular pressure above 18 mmHg or altered disk aspect such as notching, hemorrhage, thinning of the neuroretinal rim, cup to-disk ratio difference of more than 0.2 between fellow eyes and optic atrophy; diabetic retinopathy; hypertensive retinopathy; SDOCT evidence of epiretinal membranes; presence of neurological disease; and fundus autofluorescence evidence of the presence of retinal pigment epithelium (RPE) atrophy or vitelliform deposits. Each patient underwent comprehensive ophthalmoscopic examination including assessment of best corrected visual acuity (BCVA) and refractive error, slit lamp evaluation of the anterior segment, tonometry, and fundus examination. Photographic documentation of the posterior pole was performed using the photographic setting of the compass perimeter (Centervue). The evidence of SDD or CD was evaluated by two operators through the simultaneous evaluation of NIR images (Heidelberg HRT II) compared with raster images on SDOCT (Rtvue XR Avanti, Optovue, Inc, Fremont, CA, USA) according to the classification system proposed by Zweifel et al. and Spaide et al. [2,23]. Patients in the SDD group had to have evidence of at least 5 subretinal drusenoid deposits in the diameter of a papillary disc area. The presence of any CD equal to or larger than 63 μm was a reason for exclusion. Patients in the CD group had to have at least one drusen larger than 125 μm or 5 drusen between 63 and 125 μm. In order to better characterize the eyes under investigation, a quantification of the subfoveal lesions present in the two groups was made through a qualitative analysis of vertical and horizontal SDOCT scans centered on the fovea. In the SDD group 8 eyes out of 18 had subfoveal lesions (44%), while in the CD group 13 eyes out of 19 had subfoveal lesions (68%) (Figure 1). SDOCT evaluation in all patients and subjects was carried out with the following scan protocols: raster with 17 parallel-lines of standard length and width; grid with 5 vertical and 5 horizontal lines centered on the fovea; and retina map with thickness output organized in 9 Early Treatment Diabetic Retinopathy Study (ETDRS) zones, formed by circles of 1 mm, 3 mm, and 5 mm diameter centered on the fovea as defined by Gass [24]. Automated segmentation of the inner retina was carried out using Optovue software 2017.1.0.151 from the inner limiting membrane to the outer border of the IPL. Thickness values were recorded in the 9 central zones where the 3mm and 5mm diameter areas were further divided into superior, nasal, temporal, and inferior sectors [24]. Scans with quality inferior to 5/10 were rejected and re-acquired. Two expert investigators (MDP, ES) evaluated automated segmentation to check for any misalignment and in case of doubt, a senior investigator (SA) was consulted. No cases of misalignment were observed. In bilateral AMD, the choice of the eye included for analysis in the SDD and CD group was based on exclusion criteria; in the SDD and CD groups 12 and 9 fellow eyes had exclusion criteria, respectively. When both eyes in the SDD, CD, and healthy control groups could potentially be included, according to the inclusion/exclusion criteria, we used a random number generator where odd numbers were for right eyes and even numbers for left eyes. Data were expressed as mean ± standard deviation or median and interquartile range for continuous variables, and the number of cases (and percentages) for categorical variables. All variables were tested for normality using the non-parametric Kolmogorov-Smirnov test. Continuous variables were compared by the analysis of covariance (ANCOVA), adjusted for age, and a post-hoc Tukey HSD correction analysis was performed for multiple comparison analysis. Differences between means of SDD and CD group were tested using *t*-test with Bonferroni correction when required. Categorical variables were evaluated using the χ-square test or Fisher exact test when appropriate. A *p* value ≤ 0.05 was considered statistically significant.

## 3. Results

The sample included 18 eyes with SDD (SDD group), 19 eyes with CD (CD group), and 18 eyes of healthy age-matched subjects (control group). The mean age of the SDD group was 79.39 ± 8.06, the mean age of the CD group was 80.53 ± 5.35, and the mean age of the control group was 75.61 ± 6.89 years. The ages in these three groups were homogeneous and did not show statistically significant differences (*p* = 0.842). No difference in gender, BCVA, and spherical equivalent between groups was found. Demographic data are shown in Table 1.

**Figure 2:**
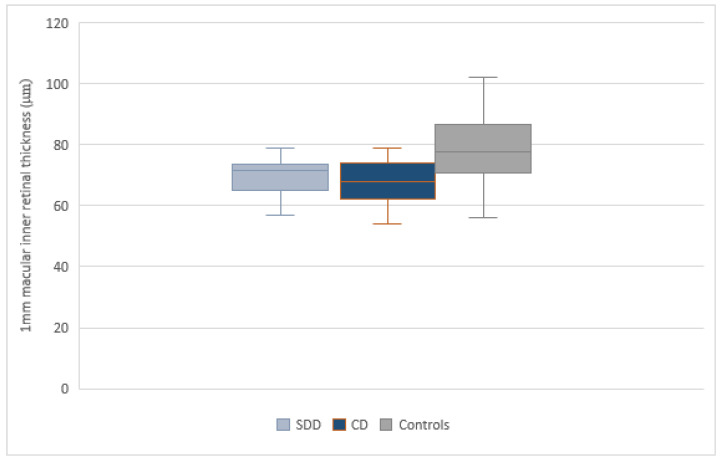
Box-plots of inner retina thickness in the central 1 mm diameter in SDD group, CD group and controls. The 1mm inner retina thickness in healthy eyes is greater than in eyes with SDD or CD. The whiskers represent the lower and upper quartile.

**Figure 3:**
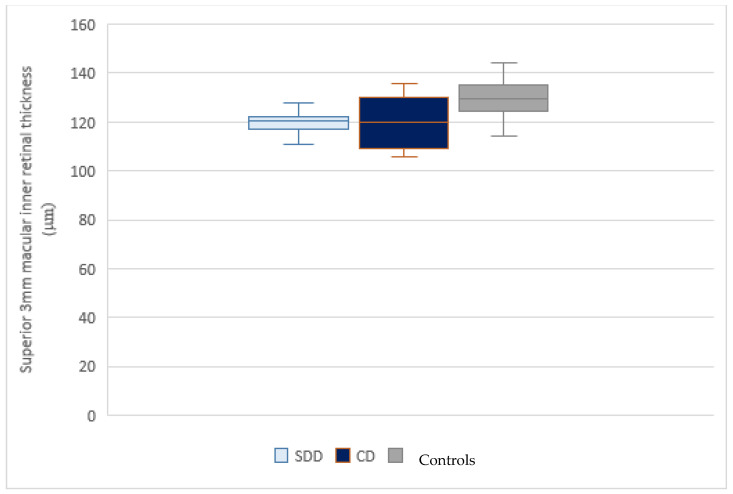
Box-plots of inner retina thickness in the superior 3 mm diameter in SDD group, CD group and controls. The superior 3mm inner retina thickness in healthy eyes is greater than in eyes with SDD or CD. The whiskers represent the lower and upper quartile.

Each group included patients with SDD only or CD only, assessed by NIR examination and SDOCT scans. Covariance analysis showed a significant reduction (*p* ≤ 0.05) in the macular inner layer thickness in the central 1 mm area in the SDD group (70 ± 6.10 µm) and in the CD group (69.21± 9.72 µm) compared to controls (78.22 ± 11.31 µm) (Figure 2); while no difference was detected between the SDD and CD group. A significant reduction of inner retinal thickness was detected in the superior sector within 3 mm above the fovea, in the SDD group and in the CD group compared to controls (*p* = 0.003) (Figure 3). No other statistically significant differences were found in the analysis in the inferior, temporal, and nasal areas at 3 mm and in the superior, inferior, temporal, and nasal areas at 5 mm between the SDD and CD groups, and between these groups with respect to controls. In order to assess the direct influence of the presence of SDD or CD on the inner retinal thickness, a separate comparison between the SDD group and control group and between the CD group and control group was also performed. Inner retinal thickness in the SDD group compared to the control group was significantly reduced in the central 1mm circle area (*p* = 0.026) and in the superior sector of the 3 mm area (*p* = 0.002). Similar results emerged in the comparison analysis between the CD group and control group. All results are summarized in Table 2.

## 4. Discussion

The current study showed that the inner retina was thinner in the central macula and superior parafoveal area in early/intermediate AMD in eyes with SDD or conventional drusen with respect to age-matched control eyes.

AMD is a progressive disease that has traditionally been regarded as an outer retinal disorder [4,5]. However, some authors evaluated inner retinal thickness by measuring the RNFL, GCL, IPL, and GCC and showed thinning of the inner retina [5,6,7,8,10,25]. In these studies topographical macular areas of evaluation, SDOCT instruments, the specific layers studied, and the classification of early or intermediate AMD were not uniform and varied widely, direct comparisons are therefore limited [5,6,7,8,10,25]. In large measure our results are consistent with these reports as we demonstrated that the inner retina is thinner in early stages of AMD.

In performing sectorial analysis we found thinning of the central and superior macular areas in eyes with early AMD with respect to control eyes. The functional and structural differences between the superior and inferior retina was reported by Curcio et al. who showed histological evidence that the retinal ganglion cell and rod density in the superior retina was higher than the inferior retina [26]. In in vivo studies, regional macular thickness alterations have been linked to microvascular changes using OCTA [6,7,18]. Trinh et al. reported a significant decrease in the superficial capillary plexus (SCP) retinal vessel density of AMD eyes, particularly in the superior quadrant [6]. Ozcaliskan et al. found a decrease in the parafoveal SCP density in eyes with intermediate AMD, in particular in the parafoveal superior areas. These authors also correlated thickness measurements with vascular density values and found that the mean parafoveal GCC was significantly correlated with parafoveal SCP vessel density [7]. These results are in line with the present study where we found thinning in the superior macular sectors. Lee et al. reported an annular pattern of parafoveal thinning of the GCL-IPL [25], and in a previous publication we reported a thinner GCL-IPL layer in the 3 and 6 mm EDTRS areas [20]. Hypotheses for this pattern could be associated with the anatomical structure of the macula that physiologically thins towards the fovea [27], the different topographic distribution of photoreceptors [7] or the vascular supply characteristics of the foveal versus the parafoveal region [8,11].

Etiopathogenetic mechanisms proposed for inner retinal involvement in AMD have been linked to trans-neural degeneration where post-receptorial functional loss due to photoreceptor damage leads to synaptic impairment [6]; this would also involve the outer layer and outer nuclear thickness and possibly the amacrine and horizontal cells. Other mechanisms include neurodegenerative disorders where inflammation and neuronal loss are the main features [1]; and microvascular changes where chronic ischemia could cause initial damage to the ganglion and inner plexiform layers cells [14], suggested even by OCTA reports on alterations of the retinal microvascular plexus [6,7,28]. Furthermore, ageing and systemic risk factors for AMD such as hypertension, atherosclerosis, and smoking may also contribute to microvascular alterations of the retina [29,30,31].

While CD are commonly, prevalently localized in the central subfield, only few SDD can be observed in this area. As described by Curcio et al. in histologic cases, SDD are found preferentially in the perifoveal area, especially in the upper sector followed by the nasal and temporal sectors, involving the foveal region in only 9% of cases [3]. For this reason, the study of retinal thickness in the central 1 mm area in patients with SDD is currently poorly evaluated, leaving room for a more in-depth study of the regions most affected by their anatomical distribution. Recently Chiang et al. carried out a study of the retinal macular thickness (including the central 1 mm area) in patients with SDD, patients with various degrees of AMD, and a control group. Their study of the inner retina showed a trend of retinal thickness reduction in the central 1 mm area in patients with SDD compared to controls [32]. Our study showed a reduction of the inner retina thickness in the central 1 mm area in patients with CD and in patients with SDD compared to controls. Thus, while our finding of thinning of the inner retina in the 1 mm central subfield is plausible in the CD group, it is rather surprising to find this in the SDD group as well. This result could be explained by the particular characteristics of our sample where a careful analysis of SDOCT scans showed a prevalence of 44% of foveal SDD. Another reason might be that in excluding eyes with mixed drusen types and selecting eyes with pure SDD, these eyes exhibit abundant drusenoid deposits including the 1mm foveal region. SDD distribution has been reported to be preferentially in perifoveal areas where there is high rod density [3]. However, of note is that in the fovea the average horizontal diameter of the rod-free zone is 0.350 mm, whereas we evaluated a larger horizontal diameter of 1 mm in the foveal region where rods can be found. We may speculate that the position of SDD in the foveal region could “mimic” the behavior of CD resulting in a thinning of the central region, similar to what happens in the parafoveal and perifoveal areas. It would be particularly interesting to determine whether inner retinal layer thinning is dependent on the presence of foveal 1 mm area and superior 3 mm area lesions, however, in our study there was not enough statistical power to stratify data.

In the present study there was no significant difference in inner retinal thickness values between eyes in the CD and SDD groups. In a previous study we reported that eyes associated with SDD had a significantly thinner GCL-IPL layer with respect to those with CD alone [20]. However, the investigation compared eyes with CD alone to those with CD associated with SDD, without a pure SDD group. Furthermore, the analysis in our previous study was different from the present report where the inner retina analysis includes the RNFL. To the best of our knowledge there is only one other study in the literature by Cicinelli et al. where the authors found a thinner GCL in eyes with SDD compared to those with CD [19]. However, Cicinelli et al. did not find any difference in RNFL values and suggested that macular RNFL could be spared as they are the innermost nerve fibers and are not initially involved in the process of trans-neuronal retrograde degeneration. Moreover, compensatory proliferation of Müller and horizontal cells could also contribute to maintaining RNFL thickness [33]. This could partially explain our results as in the present paper inner retinal analysis included the RNFL. We could not directly compare our data with the previous reports where RNFL and ganglion cell layer thickness were evaluated with different SDOCT devices that enabled to measure these layers separately. SDOCT Optovue software technology does not allow separate segmentation of the RNFL and ganglion cell layer.

Our study had some limitations. Although, it would be interesting to evaluate thickness measures for the outer nuclear layer in each of the three groups in order to possibly find correlative data for the restricted thinning of the inner retina, Optovue software technology limitations do not allow individual retinal layer thickness segmentation. Therefore, our study design was specifically focused on inner retinal thickness alterations. The number of enrolled patients and subjects is small, however, the patients had pure SDD or CD, whereas other studies also evaluated patients with mixed drusen types. The identification of SDD was performed evaluating NIR images and structural SDOCT scans obtained by two separate devices. The Rtvue XR Avanti Optovue device provides a map of the thicknesses organized in ETDRS circles of 1, 3, and 5 mm diameter as defined by Gass [24] centered on the fovea that are not the conventional 1, 3, and 6 mm circular areas studied by other authors.

In conclusion our findings suggest that there is damage to the inner retinal layers in early AMD based on structural thinning of the inner retina. Our study shows that the presence of SDD causes thinning of the inner retinal layers in the superior parafovea and the central 1mm area given the high prevalence of central SDD in our sample. Evaluation of the difference in thickness between eyes with SDD and CD requires further study on larger patient populations. An interesting line of research is the correlation of structural thickness values with microvascular retinal alterations through OCTA as vascular involvement in SDD is well known to involve the choroid that is thinner. Because vascularized retinal layers correlate positively with choroidal thickness in normal eyes [31], a conjunct evaluation of choroidal with retinal vascularity is reasonable to better understand disease pathophysiology and possible biomarkers for AMD progression and this is the endpoint of an ongoing prospective study.

## Figures and Tables

**Table 1:** Demographic data of patients with subretinal drusenoid deposits (SDD), conventional drusen (CD), and control group.

	SDD (*n* = 18)	CD (*n* = 19)	*p*-Value ^a^	Control Group (*n* = 18)	*p*-Value ^b^
Age (Years)	79.39 ± 8.06	80.53 ± 5.35	0.614 ^c^	75.61 ± 6.89	0.842 ^e^
Sex (M/F)	13/5	9/10	0.183 ^d^	7/11	0.113 ^d^
BCVA (logMAR)	0.85 ± 0.19	0.86 ± 0,17	0.868 ^c^	0.92 ± 0.11	0.331 ^e^
Spherical Equivalent (Diopters)	0.11 ± 1.40	−0.13 ± 0.89	0.533 ^c^	0.04 ± 0.1	0.780 ^e^
Phakic/Pseudophakic	13/5	12/7	0.721 ^d^	12/6	0.840 ^d^

BCVA: best-corrected visual acuity, LogMAR: logarithm of the minimal angle of resolution. a: Comparison of eyes with SDD versus eyes with CD. b: Comparison of eyes with SDD, eyes with CD and control group eyes (control group). c: T-test with Bonferroni correction. d: Fisher’s exact test. e: ANOVA.

**Table 2:** Analysis of inner retinal thickness in eyes with subretinal drusenoid deposits (SDD), conventional drusen (CD), and control group. Thickness values are expressed in µm.

	SDD (*n* = 18)	CD (*n* = 19)	*p*-Value ^a^	Control Group (*n* = 18)	*p*-Value ^b^	*p*-Value ^e^	*p*-Value ^f^
Central 1mm	70 ± 6.10	69.21 ± 9.72	0.964 ^c^	78.22 ± 11.31	0.004 ^d^*	0.026 ^c^*	0.013 ^c^*
Superior 3 mm	118.83 ± 8.99	119.89 ± 9.97	0.935 ^c^	129.55 ± 8.58	0.003 ^d^*	0.002 ^c^*	0.007 ^c^*
Inferior 3 mm	119.88 ± 10.49	117.52 ± 13.92	0.814 ^c^	123.11 ± 10.07	0.747 ^d^	0.683 ^c^	0.324 ^c^
Temporal 3 mm	114.72 ± 9.62	114.26 ± 11.77	0.448 ^c^	119.50 ± 10.45	0.472 ^d^	0.372 ^c^	0.306 ^c^
Nasal 3 mm	117.44 ± 8.44	118 ± 10.10	0.986 ^c^	124.44 ± 12.69	0.178 ^d^	0.120 ^c^	0.164 ^c^
Superior 5 mm	101.61 ± 10.12	103.21 ± 8.46	0.854 ^c^	106.88 ± 8.49	0.400 ^d^	0.191 ^c^*	0.441 ^c^
Inferior 5 mm	100 ± 6.12	98.47 ± 10.69	0.860 ^c^	102.16 ± 8.89	0.814 ^d^	0.738 ^c^	0.419 ^c^
Temporal 5 mm	99.88 ± 10.74	99.52 ± 12.71	0.994 ^c^	105.33 ± 10.73	0.572 ^d^	0.329 ^c^	0.283 ^c^
Nasal 5 mm	105.66 ± 10.92	110.57 ± 11.33	0.460 ^c^	112.38 ± 14.69	0.306 ^d^	0.238 ^c^	0.898 ^c^

a: Comparison of eyes with SDD a Comparison of eyes with SDD versus eyes with CD. b: Comparison of eyes with SDD, eyes with CD, and control group eyes (control group). c: Post hoc Tukey Honest Significant Difference (HSD), multiple comparison between groups. d: Analysis of Covariance (ANCOVA), adjusted for age. e: Comparison of eyes with SDD and control group eyes (control group). f: Comparison of eyes with CD and control group eyes (control group). * Statistically significant (*p* < 0.05).

## Data Availability

Data are available on request.
